# A validation study of the psychometric properties of the Groningen Reflection Ability Scale

**DOI:** 10.1186/1472-6920-14-214

**Published:** 2014-10-10

**Authors:** Nina Bjerre Andersen, Lotte O’Neill, Lise Kirstine Gormsen, Line Hvidberg, Anne Mette Morcke

**Affiliations:** Centre for Medical Education, Aarhus University, Aarhus, Denmark; The Research Unit for General Practice, Department of Public Health, Aarhus University, Aarhus, Denmark

**Keywords:** Assessment, Instrument, Reflection, Undergraduate medical education, Validation

## Abstract

**Background:**

Reflection, the ability to examine critically one’s own learning and functioning, is considered important for ‘the good doctor’. The Groningen Reflection Ability Scale (GRAS) is an instrument measuring student reflection, which has not yet been validated beyond the original Dutch study. The aim of this study was to adapt GRAS for use in a Danish setting and to investigate the psychometric properties of GRAS-DK.

**Methods:**

We performed a cross-cultural adaptation of GRAS from Dutch to Danish. Next, we collected primary data online, performed a retest, analysed data descriptively, estimated measurement error, performed an exploratory and a confirmatory factor analysis to test the proposed three-factor structure.

**Results:**

361 (69%) of 523 invited students completed GRAS-DK. Their mean score was 88 (SD = 11.42; scale maximum 115). Scores were approximately normally distributed. Measurement error and test-retest score differences were acceptable, apart from a few extreme outliers. However, the confirmatory factor analysis did not replicate the original three-factor model and neither could a one-dimensional structure be confirmed.

**Conclusions:**

GRAS is already in use, however we advise that use of GRAS-DK for effect measurements and group comparison awaits further review and validation studies. Our negative finding might be explained by a weak conceptualisation of personal reflection.

## Background

The ability to reflect is frequently referred to in the medical education literature and regarded as important in pre- and postgraduate medical curricula [1]. For example, it is held to be of importance in personal learning plans [2], self-critique [3], technology-mediated teaching [4], case-solving [5], clinical reasoning [6], professionalism [7], and patient safety [8]. The attempts to implement reflection and reflective practice as educational tools have been followed by a focus on assessing reflection over the last decade [9–11]. The general assumption is that students do not adopt reflective learning habits spontaneously [3], and it is often a quite difficult activity to elicit [12–15]. Furthermore, with this assessment focus, comes the need to measure reflection with the necessary degree of reliability and validity [12, 16–18]. In conclusion, reflection is important, but it can prove a difficult concept to both operationalise and measure.

In order to assess reflection, researchers need a clear concept of what reflection is. Reflection is a metacognitive process which allows the individual to learn from past experiences [19], but what does this indicate? One researcher, who has worked intensively with the different meanings of reflection in medical education, is Aukes [20]. He proposed that there are three types of reflection in the context of medical education: clinical reasoning, scientific reflection, and personal reflection. *Clinical reasoning* is defined as a "problem and patient-oriented understanding, judgment, and decision, with the key function of problem solving". It is a cognitive-logical form of reflection, which starts from an individual case. Aukes referred to *scientific reflection* as "the critical appraisal of literature and own practice", which rises above the level of an individual case. *Personal reflection* differs from the first two in being cognitive-emotional, defined by Aukes as "reflective attention to the process of sense-making in medical practice, and to the dynamics of rational and irrational thoughts and emotions, assumptions, and beliefs in that process". He concluded that the three types of reflection should co-exist in medical education, and that personal reflection should create a basis for professional functioning.

To enable the investigation and measurement of personal reflection, Aukes and colleagues developed the Dutch Groningen Reflection Ability Scale (GRAS), an instrument measuring the self-reported personal reflection ability of medical students [21]. Personal reflection was reported to consist of three underlying factors: self-reflection, empathic reflection, and reflective communication. GRAS has been used to measure the effect of an experiential learning programme and it is referred to as a scale that measures student reflection [22, 23]. To the best of our knowledge, it has not been validated since it was originally developed. Validation is a very important, but often overlooked step when using a scale in a new research setting [24].

The aim of this paper was to adapt GRAS for use in a Danish setting and to investigate the psychometric properties of GRAS-DK.

## Methods

### Ethics, context, and participants

The Danish Research Ethics Committee System does not approve or disapprove educational survey research by law. Aarhus University, Faculty of Health Sciences, approved the protocol. Data was collected, analysed, and stored according to the Danish Data Protecting Agency recommendation. Participants cannot be identified from the material. Participation was voluntary.

The research was conducted among medical students at Aarhus University. The 6-year undergraduate medical program admits students direct from secondary school. The programme is divided into a 3-year pre-clinical part (bachelor’s degree) and 3-year clinical part (master’s degree). Reflection as an educational tool within the curriculum is currently being implemented in the clinical years as portfolio assignments, but reflection was not explicitly taught at the time of the study.

The study administration at Aarhus University routinely allocates medical students into groups of approximately 25 students, who take lessons together during each semester. We sampled students in clusters based on these groups and compiled complete student lists of two randomly selected groups from each of the 12 semesters. All students in these sampled groups were invited for inclusion in the study. In other words, we followed the cluster sampling method as described by Babbie [25]. The sampling resulted in 523 students in the sample, representing all semesters, apart from two. Eighth and tenth semester students were excluded, because scheduled clinical placements made them inaccessible to the researchers. We chose to cluster sample for two reasons. Firstly, to make it feasible to compile exhaustive lists of the students in the sample, and secondly, to sample the students in existing groups, so that all included students could be visited for oral information on the study.

### Instrument

GRAS consists of 23 items measured on 5-point Likert scales with scores ranging from totally disagree (1) to totally agree (5) [21]. Individual item scores can be summed up to a total GRAS score ranging from 23 – 115. Five items (items 3, 4, 12, 17, and 21) are differently worded or negated, so that they should be reversed when scored. GRAS is administered on a single page with a set of instructions. The information, which cues participants to respond, is limited to ‘how you learn and function in practice?’ The instrument says "Learning and functioning as a medical student" and the word "reflect" or "reflection" is not mentioned.

### Cross cultural adaptation

**Figure 1:**
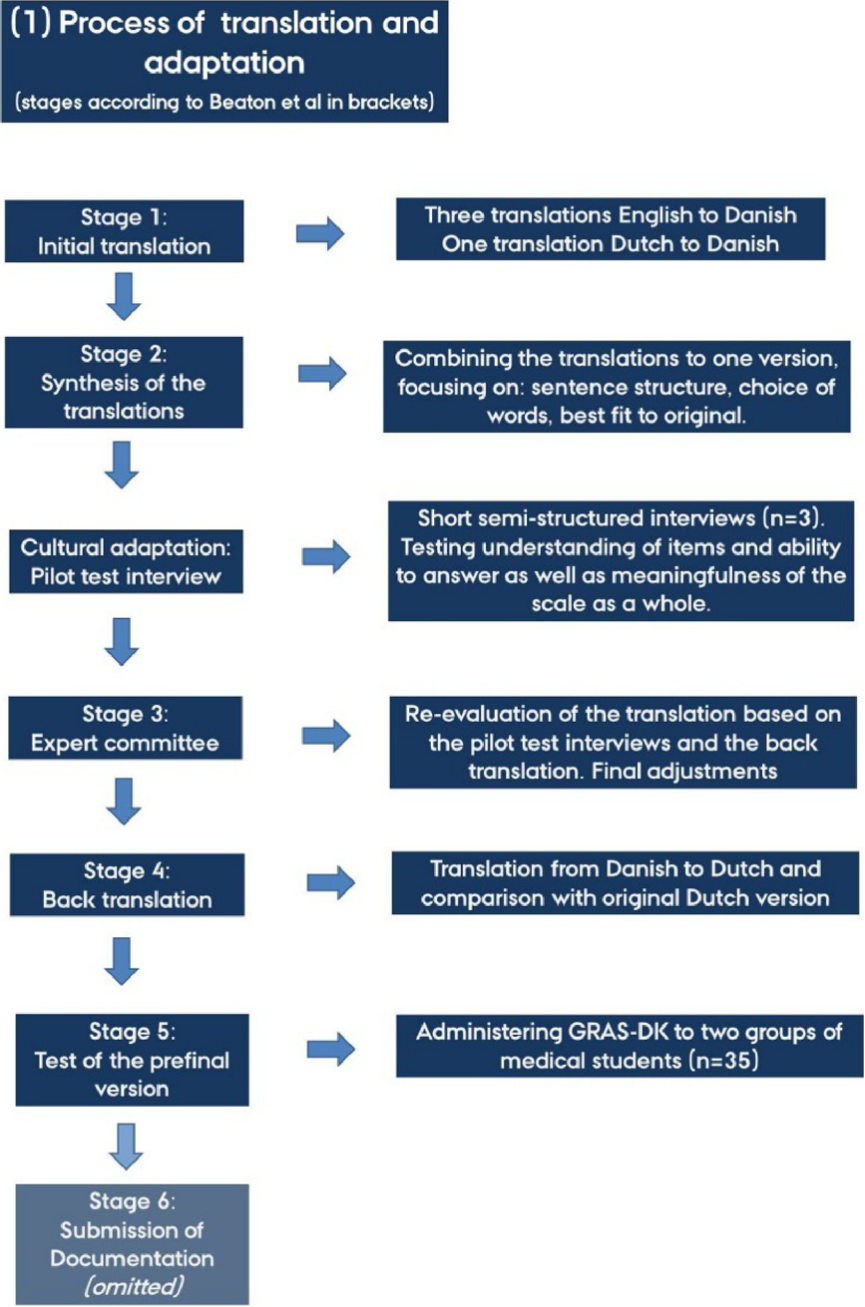
**Process flow chart.** The flow chart shows the translation and cultural adaptation process leading to GRAS-DK following the stages suggested by Beaton and colleagues [26].

GRAS exists in Dutch and an English translation [21]. Using the Dutch version, we followed the process of translation and adaption suggested by Beaton and colleagues [26] (Figure 1). In stage 1, one expert translated the Dutch version into Danish. Two other independent experts and one author also translated the English version into Danish. In stage 2, we compared the four translations and synthesised a single Danish version of GRAS, solving discrepancies by consensus.

After stage 2 (Figure 1), we conducted semi-structured pilot test interviews with three medical students, chosen by gender (two females, one male) and program year (one 7^th^ semester, 8^th^ semester, and 12^th^ semester student) on wording, translation, relevance, understanding, and time consumption. The results from the pilot test interviews were used to modify the existing version and produce a pre-final Danish version of GRAS. In stages 3 and 4, a back translation from the pre-final Danish version to Dutch tested its comparability against the original version, which did not lead to any alterations.

In stage 5 (Figure 1), we pilot tested the pre-final Danish version on two randomly selected groups of medical students (n = 35) to ensure that the electronic distribution, administration, and reminder procedure functioned well, and that the data output was usable. The final version was named GRAS-DK.

### Additional background variables and an open ended comment box

As we intended to explore arguments for validity, we added a number of background variables to the beginning of the questionnaire. The following variables could potentially be associated with a student’s reflection ability: age, gender, study year, extracurricular activity, and choice of electives. Age, gender, study year, and extracurricular activity could be analysed using descriptive statistics without further transformation of data, but choice of elective needed an additional step before our statistical analysis. In this setting students could choose between 7 different electives. Two authors (NBA and AMM) attributed each of the 7 electives a value from 1 to 4 based on a simple coding using the Structure of the Observed Learning Outcome (SOLO) taxonomy [27]. The verbs used in the learning outcomes of each of the 7 elective course descriptions elicited the value of either: 1) Uni-structural, 2) Multi-structural, 3) Relational and 4) Extended abstract. The two authors coded all 7 electives separately and reached consensus in case of discrepancies. The value of the electives could then be included in the descriptive statistical analysis.

Finally, we included an open ended question asking for comments. This is known to increase willingness to respond, resulting in a higher response rate. Further, comments from respondents can be useful in the discussion of the validity of a questionnaire [28].

### Survey administration and retest

We used the web-based instrument distribution service Survey Monkey to collect data during February 2012. One author (NBA) visited all groups in the sample, gave a verbal introduction to the survey, and subsequently invited all students in the sampled groups by e-mail. A reminder was send to non-respondents.

Approximately one month (with a variance of three days) after their first response, we invited half of the responding students to participate in a retest. We presented them with two questions to check their eligibility for retest: Have you changed how you learn and/or function in practice? We wanted to make sure that students, who might have a reason to answer differently within the months’ time, were excluded. Students that answered "no" to both questions entered the retest.

### Statistical analysis

We assumed an interval level of the data, i.e. that the difference between scores of the Likert-scale is equal along the scale based on a normal distribution of the GRAS-DK scores and used parametric statistical methods. Then, we performed descriptive statistical analyses on study population characteristics, background variables and GRAS-DK scores using StataIC 11® and examined variables for co-linearity before the definite stage of analysis by inspecting matrix graph plots and boxplots. Variables predicting GRAS-DK scores were then examined with univariate logistic regression analyses, taking p <0.1 as the criterion for inclusion in a multivariate model. We assessed the internal consistency using Cronbach’s alpha together with an assessment of potential floor and ceiling effects. The data from the first data collection were combined with the retest data to generate a Bland-Altman plot.

We conducted a confirmatory factor analysis (CFA) to test for the three factor model proposed by Aukes and colleagues [21] using the goodness of fit parameters: Comparative Fit Index (CFI), the Tucker Lewis Index (TLI), the Root Mean Square Error of Approximation (RMSEA) and the Weighted Root Mean Square Residual (WRMR) [29]. After the model had been rejected, we conducted an exploratory factor analysis (EFA) as a principal component analysis using an eigenvalue-one procedure with varimax rotation to investigate alternative item structures. This approach optimizes interpretation in terms of the correlations between each item and each factor. Items with a factor loading of 0.4 or more were assigned to a specific factor. We used M-Plus 4 to perform both factor analyses.

**Figure 2:**
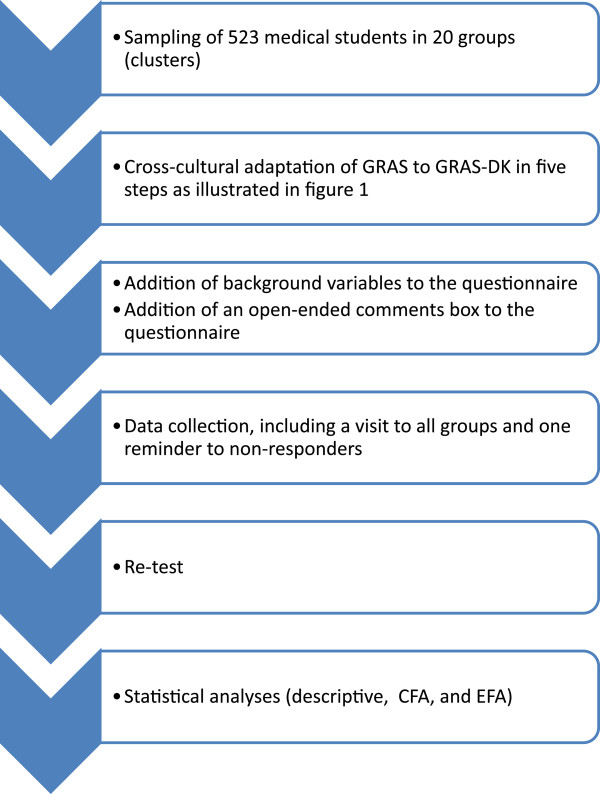
**Overall validation process.** The flow chart shows the overall validation process from sampling to the statistical analyses.

The overall validation process is shown in Figure 2.

## Results

GRAS-DK was completed by 361 (69%) of the invited 523 students. There was no significant difference between respondents and the general student population for gender and age (Table 1). Twelve participants, who did not complete the entire GRAS-DK, were excluded.

**Table 1:** **Respondents and general student population**

	Respondents	General student population
	(n = 361)	(n = 2511)
Male	124 (34%)	894 (36%)
Female	237 (66%)	1617 (64%)
Mean age (years)	24,0	23,9
SD	2.89	3,03
Min age	20	19
Max age	42	45

The table compares respondents and the general student population concerning gender and age.

**Figure 3:**
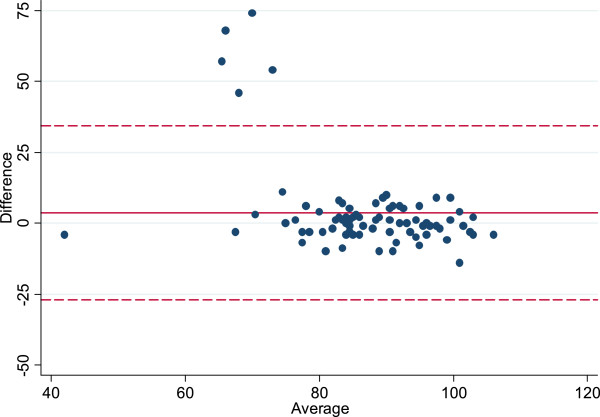
**Bland-Altman plot.** The Bland-Altman plot shows the average test score plotted against the difference between the test and retest average scores.

The mean GRAS-DK score was 88 (SD = 11.42). The scores were normally distributed apart from a small group of younger female outliers (n = 12) scoring between 40 and 55. Cronbach’s alpha was 0.87 and the average inter-item covariance was 0.22. The distribution of GRAS-DK scores showed no overall floor or ceiling effect. At the item level, some items had more than 40% of answers in the lowest (items 8 and 12) or highest (items 1, 19 and 22) answer categories, which represent single item floor and ceiling effects respectively.112 (65%) of the 172 students that we invited for the retest responded, and 83 of them fulfilled the inclusion criteria. Using a Bland-Altman comparison of test and retest scores, the mean difference on GRAS-DK scores was found to be 3.55 (CI: [0.21; 6.90]) with limits-of-agreement of -27.12 to 34.23 (Figure 3). Five outliers showed a high disagreements between test and retest values.

The CFA did not replicate the three factor model proposed by Aukes and colleagues [21] and the only index that showed a good fit was the RMSEA (Table 2). Table 3 shows the single item loadings on the three factors. Especially the reversed items loaded low on their respective factors, item 8 being the exception among the reversed items.

**Table 2:** **The confirmatory factor analysis**

	Results	Interpretation
CFI	0.885	0.9-0.95: Acceptable fit
TLI	0.872	>0.95: Good fit
RMSEA	0.076	≤ 0.05: Very good fit
		>0.05 < 0.10: Good fit
		≥ 0.10: Bad fit
WRMR	1.330	<0.9: Good fit

The results from the confirmatory factor analysis are based on the Comparative Fit Index (CFI), the Tucker Lewis Index (TLI), the Root Mean Square Error of Approximation (RMSEA,) and the Weighted Root Mean Square Residual (WRMR). The interpretation shows the level of index findings that would indicate a good fit of the data to the original three-factor model.

**Table 3:** **The factors loadings of the 23 GRAS –DK items**

	FACTOR 1	FACTOR 2	FACTOR 3
1. I want to know why I do what I do	0.542		
2. I am aware of the emotions that influence my behaviour	0.639		
3. I do not like to have my standpoints discussed			0.466
4. I do not welcome remarks about my personal functioning			0.374
5. I take a closer look at my own habits of thinking	0.491		
6. I am able to view my own behaviour from a distance	0.553		
7. I test my own judgments against those of others	0.384		
8. Sometimes others say that I do overestimate myself		0.418	
9. I find it important to know what certain rules and guidelines are based on	0.398		
10. I am able to understand people with a different cultural/religious background		0.639	
11. I am accountable for what I say			0.863
12. I reject different ways of thinking		0.612	
13. I can see an experience from different standpoints	0.739		
14. I take responsibility for what I say			0.810
15. I am open to discussion about my opinions			0.749
16. I am aware of my own limitations		0.472	
17. I sometimes find myself having difficulty in illustrating an ethical standpoint			0.157
18. I am aware of the cultural influences on my opinions	0.559		
19. I want to understand myself	0.677		
20. I am aware of the possible emotional impact of information on others		0.727	
21. I sometimes find myself having difficulty in thinking of alternative solutions			0.203
22. I can empathize with someone else’s situation		0.727	
23. I am aware of the emotions that influence my thinking	0.733		

The table lists the confirmatory factor analysis factor loadings of the 23 GRAS-DK items.

The EFA, which included trying a set three factor model as well as leaving items with consistent low loadings out of the analysis, produced a diffuse distribution of loadings that did not conform to a one-dimensional model. We concluded from this that: 1) no factor model could explain enough of the variance to be a satisfactory fit, and 2) especially the reversed items seemed to function poorly in the instrument.

There was a small, statistically significant difference in GRAS-DK score of 2.58 (95% CI: 0.38; 4.78) between male and female students (89.27 vs. 86.70). Also, the few students (n = 6) who had followed an elective with the highest taxonomy level (most extended abstract learning outcomes) had a significantly higher GRAS-DK score than students who had followed the other electives. There was no correlation between GRAS-DK score and age, study progression, or extracurricular activity.

In the open ended question box where participants could freely comment, some found the scale lacking context (n = 15), with students commenting that they did not know which part of their life they should relate the items to. Others found the items very abstract and found it hard to answer questions that they had never thought about before (n = 13). The terminology used to describe reflection was also an issue for some (e.g. "habits of thinking") (n = 8). However, students also found the items "relevant" and "interesting to think about".

## Discussion

This study investigated the construct validity of GRAS-DK, its measurement error, and its content validity. GRAS-DK functioned well in a test-retest situation, apart from a few extreme outliers. The three-factor model of the original GRAS could not be reproduced, however. The 23 items of GRAS-DK did not fit into a statistical model, and GRAS-DK was not found to be a one-dimensional scale.

### Strengths and limitations

The transfer of the GRAS for use in a different international setting could have resulted in subtle differences in linguistic nuance between the original GRAS and the GRAS-DK. But seeing that we followed a rigorous and systematic cross-cultural adaptation process aimed at reducing language inaccuracies, potential differences between GRAS and GRAS-DK are most likely minor, and we find it unlikely that this alone could explain the lack of confirmation.

The response rate was 69%, which is acceptable and markedly higher than the average for electronic questionnaires [30]. To enhance the response rate, participants were cluster sampled according to their group affiliation, because this enabled us to do personal group introductions to the survey. Cluster sampling is not the preferred way to ensure a representative sample, because individuals in a cluster can be similar due to their common cluster affiliation. In this study, there is no reason to believe that the student groups were more similar within groups than across the groups. The study population corresponded to the general student population on selected background variables, indicating that the respondents were most likely a representative sample.

### Validity

According to the latest (1999) Standards for Educational and Psychological Testing, validity refers to the degree to which evidence and theory support the interpretations of test scores entailed by the proposed uses of tests. Thus the process of validation involves accumulating evidence to provide a sound scientific basis for the proposed score interpretation [31]. Since the old trinitarian model has been replaced with a unified validity framework, the discourse in modern validity theory has moved from examining types of validity (content, criterion, construct) to examining sources of validity evidence (including evidence of consequences of test interpretation and use), which are all seen as counting towards construct validity [31, 32]. In other words, although there may be different sources and mixes of validity evidence for supporting the adequacy and appropriateness of interpretations of scores in different situations, validity is seen a unitary concept in modern validity theory [33].

Validity should be tested through solid, logical arguments to support that an instrument actually measures what it purports to measure [34]. Kane described how validation involves an interpretative argument, i.e. a specification of the proposed test interpretation and use, and a validity argument which provides an evaluation of the interpretative argument [32]. Interpretative arguments typically include major inferences relating to: scoring, generalization, extrapolation, theory based interpretation (for theoretical constructs), and decisions/implications. In the following, we shall go through relevant inferences that relate to our findings and discuss the validity of GRAS-DK based on these inferences.

The first inference relates to the scoring process: GRAS-DK is a self-report measure scored on a 5-point Likert scale and the difficulty for the respondents to answer some items hints that not all items may correspond to a meaningful score. We suggest that a reason for this could be that the items are not grounded in a context where respondents know what they should refer to when answering. Also, we suggest that some items in the instrument could be too vaguely formulated ("I want to know why I do what I do") and some too concrete ("Sometimes others say that I do overestimate myself") in order for students to understand how they should be scored. Furthermore, we wonder, whether a high score indicates a high level of reflection. For example, is it a measure of high personal reflection to agree that one understands people of a different cultural background – or is it reflective to rather be self-critical and indicate that it is challenging to understand other people? It has been argued that self-report measures face this exact issue of validity, because it can be hard to distinguish whether it is reflection or the ability to introspect that is being measured [17].

The second inference concerns generalization (i.e. going from observed score to a universe score): We evaluated the test-retest properties of GRAS-DK at the universe score level. GRAS-DK proved to have an acceptable measurement error, although five extreme outliers impact the Bland-Altman limits-of-agreement greatly. We do not know the reason for these few respondents skewing the picture, but they affect individual and group level measurements alike. Furthermore, we note that the responsiveness*,* indicating whether an instrument can detect actual change in a respondent over time, has not yet been tested on either GRAS or GRAS-DK.

The third relevant inference relates to extrapolation (i.e. going from universe score to the target domain): We suggest that the major problem of GRAS-DK and possibly GRAS lies here; in the connection between the definition of personal reflection and the scale. Validity here refers to whether the items of a scale comprehensively represent the concepts of interest [35]. The failure to reproduce the proposed three-factor model and thereby support a one-dimensional scale is a strong argument against GRAS-DK’s validity. As Schuwirth & Van der Vleuten concluded: "the arguments of validation can only be made if the construct we want to assess is defined clearly enough and when all theoretical notions about it are sufficiently concrete" [34]. We conclude that this might not be the case with GRAS and GRAS-DK. Our study is not alone with a negative or limited finding in a study measuring medical students’ reflection [13–15, 36, 37], and we call for further research on the construct ‘personal reflection’.

We recommend that personal reflection ability is further clarified in order for it to be operationalized. Furthermore, we find that GRAS-DK should not be used for effect measurements and group comparisons before the instrument has been revised for conceptual clarification of the content validity of the items. In order to numerically measure reflection to show the effects of educational interventions or follow student development over time, the instrument needs further validation and development to meet the necessary quality criteria.

## Conclusions

GRAS-DK, a Danish version of GRAS, did not function well as a measure of personal reflection. GRAS-DK could not be interpreted as one scale, the original three-factor model was not confirmed, and a weak conceptualisation is proposed to be the major problem. This conclusion should by no means lead to the conclusion that we do not find reflection important. On the contrary, we agree with Mann and colleagues [1] and hold reflection to be very important to medical education and doctors. The conceptualisation of reflection for practical use in teaching and assessment seems quite difficult, despite the good work of other researchers. Thus, the international solution to assessing reflection levels among medical students is not found, but the evidence-based discussions hopefully continue with underlying positive – as well as – negative findings.

## Authors’ information

NINA BJERRE ANDERSEN, BSc, is a 5^th^ year medical student and junior researcher at the Centre for Medical Education, Aarhus University, Denmark. In 2012 she received a travel grant from Her Majesty Queen Margaret II for her activities and accomplishments. Nina’s research interests include reflective learning, medical humanities, and narratives.

LISE KIRSTINE GORMSEN, PhD, MD, MHH, is an assistant professor at the Centre for Medical Education, Aarhus University, and a resident doctor at Aarhus University Psychiatric Hospital, Denmark. Lise’s research interests include ‘the good doctor’ and the role of humanities in medical education with a particular focus on fictional literature.

LOTTE O’NEILL, PhD, MMedEd, MSc, is an assistant professor at the Centre for Medical Education, Aarhus University, Denmark. Lotte’s research interests include admission tests, drop out in higher education, and students and residents in difficulties.

LINE HVIDBERG, MSc, is a PhD student at the Research Unit for General Practice, Department of Public Health, Aarhus University, Denmark. Line’s research interests include patient behaviour and perceptions of illness with a focus on differences in cancer awareness and beliefs, and patients’ notions of cancer symptoms and screening.

ANNE METTE MORCKE, PhD, MD, is an associate professor at the Centre for Medical Education, the director of undergraduate medical education, and chair of the medical curriculum committee at Aarhus University, Denmark. Her current research interests include curriculum theories as the basis for sound curriculum development that promote student learning.

## Abbreviations

CFA: Confirmatory factor analysis
CFI: Comparative Fit Index
EFA: Exploratory factor analysis
GRAS: Groningen Reflection Ability Scale
GRAS-DK: Groningen Reflection Ability Scale Denmark
RMSEA: Root Mean Square Error of Approximation
SOLO: Structure of the Observed Learning Outcome
TLI: Tucker Lewis Index
WRMR: Weighted Root Mean Square Residual.
